# Phenotypic Trait Identification Using a Multimodel Bayesian Method: A Case Study Using Photosynthesis in *Brassica rapa* Genotypes

**DOI:** 10.3389/fpls.2018.00448

**Published:** 2018-04-17

**Authors:** Jonathan R. Pleban, D. Scott Mackay, Timothy L. Aston, Brent E. Ewers, Cynthia Weinig

**Affiliations:** ^1^Department of Geography, University at Buffalo, Buffalo, NY, United States; ^2^Department of Botany, University of Wyoming, Laramie, WY, United States; ^3^Program in Ecology, University of Wyoming, Laramie, WY, United States; ^4^Department of Molecular Biology, University of Wyoming, Laramie, WY, United States

**Keywords:** *A/C*_*i*_ curves, Bayesian models, *Brassica rapa*, chlorophyll fluorescence, multimodel analysis, phenotyping, photosynthesis

## Abstract

Agronomists have used statistical crop models to predict yield on a genotype-by-genotype basis. Mechanistic models, based on fundamental physiological processes common across plant taxa, will ultimately enable yield prediction applicable to diverse genotypes and crops. Here, genotypic information is combined with multiple mechanistically based models to characterize photosynthetic trait differentiation among genotypes of *Brassica rapa*. Infrared leaf gas exchange and chlorophyll fluorescence observations are analyzed using Bayesian methods. Three advantages of Bayesian approaches are employed: a hierarchical model structure, the testing of parameter estimates with posterior predictive checks and a multimodel complexity analysis. In all, eight models of photosynthesis are compared for fit to data and penalized for complexity using deviance information criteria (DIC) at the genotype scale. The multimodel evaluation improves the credibility of trait estimates using posterior distributions. Traits with important implications for yield in crops, including maximum rate of carboxylation (*V*_*cmax*_) and maximum rate of electron transport (*J*_*max*_) show genotypic differentiation. *B. rapa* shows phenotypic diversity in causal traits with the potential for genetic enhancement of photosynthesis. This multimodel screening represents a statistically rigorous method for characterizing genotypic differences in traits with clear biophysical consequences to growth and productivity within large crop breeding populations with application across plant processes.

## Introduction

Maintaining food security for the world's rapidly growing population is a paramount challenge for science. Classic and modern genomic breeding programs represent one of the major tools for increasing food supply to counter this Malthusian dilemma. The success of breeding programs in part depends on the ability to quickly identify beneficial phenotypic traits in breeding populations (Sadras et al., 2013). Experimentation and modeling assists trait identification while producing insights into plant physiology and crop productivity (Sinclair and Seligman, 1996; Hammer et al., 2006). Mechanistic modeling using known or theorized ecological, biochemical and biophysical principles further advance understanding through connecting yield to causal traits (Laisk and Nedbal, 2009; Tardieu, 2010). These mechanistic models of plant physiology use statistical tools to estimate trait variation by organizing phenomenological data into meaningful mathematical representations of enzymatic and protein activity responsible for plant processes (DeWitt, 1965; von Caemmerer, 2000; Patrick et al., 2009; McDowell et al., 2013). In this way models can estimate valuable phenotypic information through specifying physiologically meaningful trait values from data.

Photosynthesis is a primary target for selective enhancement in crops (Long et al., 2006; Singh et al., 2014; Furbank et al., 2015) and the mechanisms of photosynthesis are well-studied with models used to characterize assimilatory strategies across taxa (Wullschleger, 1993; Patrick et al., 2009; Gu et al., 2012). For these reasons, photosynthesis was chosen as a target for multimodel phenotyping. Modeling of photosynthesis evolves as theory tests data in attempts to replicate the pathway of enzymatic and protein responses responsible for carbon fixation, light harvesting, and electron transfer (DeWitt, 1965; Farquhar et al., 1980, 2001; von Caemmerer, 2000; Yin et al., 2009). At each stage of model development simplifying assumptions are made regarding the behavior of these pathways. For example, assumptions are made in regard to the degree of trait response to temperature fluctuation or regarding the relative resistance change in the CO_2_ pathway from the atmosphere to the site of carboxylation (Medlyn et al., 2002a; Pons et al., 2009). Such assumptions affect modeling efforts in three key ways. First, assumptions accommodate unknowns and can shift model emphases from mechanistic processes to empirical relationships. Second, assumptions impact model complexity, forcing modelers to assess the performance of models ranging in complexity (Knorr and Heimann, 2001; Martre et al., 2015). Third, assumptions can influence the uncertainty of trait estimates (Mackay et al., 2012). Uncertainty quantification is therefore necessary when making claims of trait differentiation. More broadly, uncertainty quantification can inform the cyclical process of model improvement by repeatedly testing updated theory against data (Box, 2001).

A number of theoretical developments with empirical support have identified the critical factors limiting leaf-level CO_2_ assimilation (*A*). Generally, the limiting factors are divided into two major classes: diffusional and biochemical. Diffusional limits can be further subdivided into a stomatal limitation that is imposed by guard cell control over stomatal conductance and a mesophyll limitation regulating CO_2_ and H_2_O transport between the intracellular space and the site of carboxylation (*C*_*c*_) (Ethier and Livingston, 2004; Grassi and Magnani, 2005; Niinemets et al., 2009a; Damour et al., 2010). Limits on photosynthesis imposed by mesophyll conductance (*g*_*m*_) have been shown to be of a similar magnitude to *g*_*s*_ limitation (Grassi and Magnani, 2005). However, uncertainty remains in understanding the limits *g*_*m*_ imposes across taxa and environments as all methods of estimating *g*_*m*_ rely on models sensitive to parameterization (Pons et al., 2009; Gu and Sun, 2014; Théroux-Rancourt and Gilbert, 2017). The leaf biochemical limitations controlling *A* are summarized by two primary factors, Ribulose-1,5-bisphosphate carboxylase/oxygenase (RuBisCO) limited *A* (*A*_*c*_) and regeneration of ribulose biphosphate (RuBP) limited *A* (*A*_*J*_). *A*_*c*_ follows the Michaelis–Menten enzymatic kinetics for RuBisCO. This requires amongst other parameters the estimation of the maximum rate of carboxylation (*V*_*cmax*_). *A*_*J*_ is coordinated by the electron transport rate (ETR) across photosystems II and I (PSII, PSI), which produces ATP and NADPH needed for the Calvin carboxylation cycle (von Caemmerer, 2000). Measurements of chlorophyll fluorescence have been used as proxies for ETR limited *A*_*J*_ (Genty et al., 1989; Baker, 2008). A carbon metabolism limitation or triose phosphate utilization (TPU) limitation has also been identified (Sharkey et al., 1985). Beyond the major diffusional and biochemical limitation, studies have sought to better understand the influence temperature has on photosynthetic performance (Bernacchi et al., 2001; Medlyn et al., 2002a; Patrick et al., 2009). Temperature influence can be modeled using an activation energy (*E*_*i*_) parameter following an Arrhenius function (von Caemmerer, 2000). Both diffusional and biochemical limitations are temperature-responsive using these modeling methods (Bernacchi et al., 2001, 2002; Leuning, 2002; Kattge and Knorr, 2007). In total, the inclusion or absence of these limitations, constraints or assumptions regarding leaf biochemistry and biophysics results in models of varying complexity.

Progressively, each incremental change in model form represents an alternative view of how the photosynthetic machinery behaves. A multimodel framework can test for the strengths and weaknesses of these alternative views. Information criteria such as Akaike Information Criteria and Bayesian correlate Deviance Information Criterion (DIC) provide metrics for evaluating model adequacy through combining terms of both the goodness of fit and model complexity (Akaike, 1998; Spiegelhalter et al., 2002). A recent statistical argument suggests that multimodel analyses are a superior method, compared to null hypothesis approaches, to test mechanisms against data (McElreath, 2016). Climate models leverage the multimodel approach for generating estimates of regional temperature change (Tebaldi et al., 2004) and these climate model ensembles have been used in crop prediction models (Ruane et al., 2017). Hydrological work also embraces Bayesian multimodel approaches to both inform groundwater estimates and sampling schema (Xue et al., 2014).

The current state of the art in photosynthesis models seeks to capture known and theoretical biophysical processes of the diffusional limitations and the light and light-independent reactions responsible for observed variation across environments and taxa (van der Tol et al., 2009; Yin et al., 2009). Concurrently modelers aim to parameterize at finer evolutionary scales (Yin et al., 2001; Patrick et al., 2009; Yamori et al., 2014). The broad division of models between C-3 and C-4 plants represents a critical improvement in this context (von Caemmerer, 2000). The C-3/C-4 evolutionary shift resulted in mechanistic differences requiring unique modeling frameworks for successfully understanding these two broad assimilatory systems. But approaches ignore model structural differences when considering variation among closely related individuals. Here we argue that multimodel analysis can assist in testing for variation across evolutionary context, as the structure, distribution, abundance, and therefore behavior of critical enzymes and proteins should be conserved in closely related populations relative to evolutionarily distant ones. Of relevance to crop breeding and highly outcrossing wild species, allelic combinations from two parents may lead to physiological responses beyond the range expressed by the parents due to transgressive segregation (Rieseberg et al., 1999). In these cases, identifying genetic controls over biophysical traits and processes across the species as a whole may be more complicated. Even so, instances of transgressive segregation explore phenotypic space that may be acted upon by natural or artificial selection (Rieseberg et al., 2003). In such cases genotypic differences may require genotype-specific behaviors and allow for reduced model complexity by eliminating the need for particular parameters. For example, it may be unnecessary to account for *g*_*m*_-limitation if all experimental genotypes have uniformly high *g*_*m*_ relative to *g*_*s*_. Therefore, when probing for trait variation within a genetically variable species, it is important to explicitly test if genotype- or species-level model forms are preferred. Overall, our aim was a robust complexity analysis of multiple leaf photosynthesis models for evaluation of photosynthetic differences of different allelic combinations.

## Materials and methods

### Overview

Model evaluation should not only considerer fit because increasing complexity often improves fit but may not increase predictive power. Therefore, checking models for both fit and parsimony should occur iteratively (Box, 1976; Spiegelhalter et al., 2002). To avoid unnecessarily high dimensionality, statistical measures have been developed that penalize complexity as part of a model selection strategy (Akaike, 1998; Spiegelhalter et al., 2002; Plummer, 2008). In the screening tool developed here, the complexity associated with three leaf level modifications of photosynthesis models was quantified: temperature constraints, *g*_*m*_ limitation, and derivational form of ETR.

**Figure 1:**
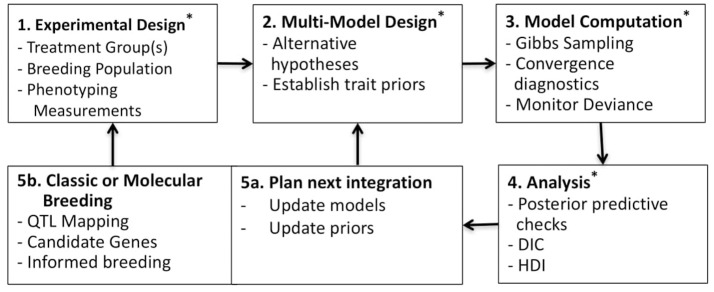
Workflow for multimodel Bayesian phenotyping. Box 1: Experimental design across varietals and/or treatments; here focus was on *A*/*C*_*i*_ curves from six *B. rapa* genotypes. Box 2: Alternative models constructed for testing assumptions regarding the process of interest; here eight photosynthesis models with trait priors established from literature (Tables 1–4). Box 3: Bayesian sampling for generating posterior distributions across all models X individuals, here executed in rjags (Plummer, 2014). Box 4: Posterior distributions evaluated using posterior predictive checks (Figures 2–4), genotypic model complexity compared using DIC (Table 5), and posterior trait distributions of each model scrutinized using boxplots (Figures 5–8) and high-density interval (HDI) analysis (Table 6). Box 5a: Model evaluation and updating along with updating of prior distributions for population. Box 5b: Genomic analysis of identified traits and classic or molecular breeding for establishing next test population (Figure 9). ^*^ indicates steps competed in this study.

Bayesian methodologies are well-suited for parameterization, uncertainty analysis and multimodel evaluation (Gelman et al., 2004; Kruschke, 2010). These methods have wide application in plant ecology and physiology (Ogle and Barber, 2008; Patrick et al., 2009; Mackay et al., 2012; Gou et al., 2017). While many Bayesian studies have focused on just one or two of these beneficial features; often parameterization and uncertainty analysis (Zhu et al., 2011; Gou et al., 2017), we sought to leverage all three features. Additionally, Bayesian methodologies can be tested in hierarchical structures whereby multiple datasets can be combined to inform parameterization at different levels, such as among taxa or genotypes, spatial scales or other known sampling characteristics (Gelman et al., 2004). Here, a simple two level hierarchical structure is adopted, where individual and genotypic level traits are estimated. This analysis is summarized in Figure 1 as a five-part trait-screening methodology; four of these five parts are presented in detail here. First, experimental data, described later in the Plant Physiological Measurements section, is collected across genotypes and/or within treatments; the data set presented here is across genotypes under unstressed growth conditions. Second, alternative models are constructed in an effort to challenge commonly held yet non-definitive assumptions regarding the process of interest; here multiple photosynthesis models are established using curves of CO_2_ assimilation (*A*) vs. intercellular CO_2_ concentrations (*C*_*i*_) (*A/C*_*i*_*)*. Third, observation data is passed into competing models using a sampling scheme designed to produce Bayesian posteriors. Fourth, the posterior distributions of each model are examined, in order to identify traits with genotypic differentiation and to evaluate genotypic differences in light of model complexity. Fifth, findings are summarized to develop future experimental-modeling iterations. In sum, potentially beneficial traits are identified, areas for model improvement are considered, and resultant posterior distributions are used to update trait priors for subsequent evaluation. At the same time, new experimentation can be considered based on findings and genetic analysis of trait estimates may support classic or molecular breeding based on candidate genes. Ultimately, this multimodel analysis aims at critically evaluating trait differences within the population under investigation and represents an important step in linking phenome to genome, here demonstrated on the vital processes of carbon assimilation and light harvesting.

### Study site and genotypes

Data were obtained from experiments undertaken in the summers of 2012 and 2013 at the University of Wyoming Research and Extension Center Field Complex (41.32 N, 105.56 W) in Laramie WY, USA. Details of the experiment are given in Aston et al. (in prep). We included six genotypes of *B. rapa*: four crop accessions [oilseed, subsp. *Pusa Kalyani* cgn06834 (*oil*); turnip, subsp. *Maiskaja* cgn06710 (*tur*); Chinese cabbage, subsp. *Pekinensis* cgn13942 (*cab*); broccoletto subsp. *Quarantina* cgn06825 (*bro*)] and two recombinant inbred lines (RILs) (*r46* and *r301*). Crop accession seeds were obtained from the Wageningen University and Research Center for Genetic Resources. The RILs, *r46* and *r301*, are the F8 offspring of a cross between the IMB211 genotype derived from the Wisconsin Fast Plant™ population and the R500 genotype, an oilseed long cultivated in India. The two RILs, full siblings, were selected based on the expression of transgressive segregation for intrinsic water use efficiency (*WUE*) identified in earlier research (Edwards et al., 2011, 2012). Plants were germinated in a greenhouse and after two weeks transplanted to a rain shelter in the field where they were grown under well-watered conditions. All measurements were taken on days 25–28 after planting.

### Plant physiological measurements

Infrared leaf gas exchange (IRGA) measurements (Li-6400XT, Li-Cor, Lincoln, NE, USA) were taken to measure *A*/*C*_i_ response with a constant irradiance of 2,000 μmol m^−2^ s^−1^ for CO_2_ concentrations of ~50, 100, 200, 300, 400, 500, 600, 800, 1,000, 1,250, 1,500, and 2,000 μmol mol^−1^. IRGA measurements monitor fluxes of both CO_2_ and H_2_O allowing for direct measurement of *A* and transpiration (*E*) with indirect means of assessing *g*_*s*_ and *C*_*i*._ CO_2_ response curves were measured between 10:00 and 16:00 h on fully expanded, mature leaves with leaf temperature maintained near 22°C and relative humidity maintained within 10% of ambient. A steady state was achieved at each CO_2_ increment prior to each gas exchange observation. Temperature and vapor pressure deficit averaged 21.1°C (±3.0) and 2.01 kPa (±0.4), respectively. Chlorophyll fluorescence measurements were taken in conjunction with each gas exchange observation. Chlorophyll fluorescence observations measure the re-radiated near infrared light by the leaf. Fluorescence serves as one means, albeit small, of dissipating excess light energy (Maxwell and Johnson, 2000). Fluorescence yield is used to quantify the amount of light energy transferred from excited photosystem II to primary quinone acceptors, such as plastoquinone, driving downstream photosynthetic light reactions (Baker, 2008). This is done using a saturating flash followed by a dark pulse to measure *F*_*m*′_ – *F*_*s*_/*F_m_′*, where *Fs* is steady state fluorescence yield and *F_m_′* is maximum light-adapted fluorescence yield. *F_m_′* –*F*_*s*_/*F_m_′* is commonly referred to as effective quantum yield of PSII (ϕ_*PSII*_*)* (Genty et al., 1989). A simple method using ϕ_*PSII*_ to estimate total flux of the electron transport chain based on fluorescence (*J*_*f*_) (μmol m^−2^ s^−1^) is described in Equation (3.5) of Table 2. Equation (3.5) makes assumptions regarding the partitioning of light between photosystems (*f*) (0.5) and the fractional value of light absorptance by leaf photosynthetic pigments (α_*leaf*_) (0.85). In total 31 individual *A/Ci* curves were tested in the analysis (six *r301*, six *r46*, five *bro*, five *cab*, three *oil*, and six *tur*).

### Bayesian modeling approach

A Bayesian model is comprised of three sets of probability statements (Gelman et al., 2004; Kruschke, 2010; Ogle and Barber, 2011). First, prior statements represent a statistically sound and repeatable method of summarizing known information, in this case regarding plant photosynthesis physiological traits (Ogle and Barber, 2008). The second probability statement is the likelihood function(s) expressing the probability that a given model could have generated a particular set of data. The final probabilistic statements are the posterior distributions describing the strength of a model once data have been tested as well as the degree of uncertainty in parameterization. Here 31 *A/Ci* curves were independently evaluated using a suite of eight models following this approach. The data, parameters, and predictions made by these eight models are described in Table 1, with model equations identified in Table 2. Each model has a coded name based on the assumptions therein (Table 3). The model and implementation codes used for analysis are provided at https://github.com/jrpleban/Bayes_Farquhar_Models_2_level_Hierarchy. Priors on parameters are shown in Table 4. We have chosen to estimate some parameters often set as constants (*K*_*c*_, *K*_*o*_) to evaluate a given model's ability to discern traits expected to be conserved in this population. A literature survey for each parameter was used to provide statistical distributions for parameter priors. Many parameters have ample data across taxa, such as for *J*_*max25*_, allowing a normal distribution of priors (Wullschleger, 1993). When a trait's distribution was more uncertain, broad priors were used, such as for ϕ_*J*_.

**Table 1:** List of abbreviations used for observations, predictions, and parameters of eight photosynthesis models.

**Abbreviation**	**Definition**	**Units**	**Models using**
**OBSERVATIONAL DATA**			
*A_*n*_*	CO_2_ assimilation rate observed	μmol m^−2^ s^−1^	All
*C_*i*_*	Intercellular CO_2_ partial pressure observed	Pa	CiCc Models
*C_*a*_*	Ambient CO_2_ partial pressure observed	Pa	CaCc Models
*T*	Leaf temperature observed	°C	Temp Models
*P*	Pressure observed	Pa	All
*g_*s*_*	Conductance to CO_2_ from atmosphere to intercellular space observed	μmol m^−2^ s^−1^	CaCc Models
*O*	Ambient O_2_ (assumed 21% atmosphere)	Pa	All
*Q*	Photosynthetically active radiation observed	μmol m^−2^ s^−1^	All
ϕ_*PSII*_	Quantum yield of photosystem II based on Chlorophyll fluorescence	e^−^ photon^−1^	Jf Models
*J_**f**_*	Electron transport rate from Chlorophyll fluorescence (Equation 3.5)	μmol m^−2^ s^−1^	Jf Models
**PROCESS MODEL PREDICTIONS**			
*A_*exp*_*	Expected CO_2_ assimilation rate	μmol m^−2^ s^−1^	All
*A_*c*_*	Rubisco limited rate of CO_2_ assimilation	μmol m^−2^ s^−1^	All
*A_*j*_*	Electron transport limited rate of CO_2_ assimilation	μmol m^−2^ s^−1^	All
*J_*m*_*	Rate of electron transport following Equation (3.6)	μmol m^−2^ s^−1^	Jm models
**PROCESS MODEL CONSTANTS**			
*R*	Universal gas constant (8.314 J K^−1^ mol^−1^)	J K^−1^ mol^−1^	All
α_*leaf*_	Absorptance of leaf photosynthetic pigments (0.85)	unitless	All
*f*	Partitioning of energy between PSII and PSI (0.5)	unitless	Jf Models
**PROCESS MODEL PARAMETERS IDENTIFIED BY BAYESIAN ESTIMATION**			
*R_*d*_ (R_*d25*_)*	Respiration rate in the dark (standardized to 25°C)	μmol m^−2^ s^−1^	All
Γ^*^ (Γ^*^25)	CO_2_ photocompensation point (standardized to 25°C)	Pa	All
*K_*c*_ (K_*c25*_)*	Michaelis-Menten constant for Rubisco for CO_2_ (standardized to 25°C)	Pa	All
*K_*o*_ (K_*o25*_)*	Michaelis-Menten constant for Rubisco for O_2_ (standardized to 25°C)	kPa	All
Ei's (_*Kc*_, _*Ko*_, _*Rd*_, _*Vcmax*_, Γ^*^, _*Jmax*_, _*gm*_)	Activation energy used in Arrhenius function	KJ mol^−1^	Temp Models
*g_*m*_ (g_*m25*_)*	Mesophyll conductance to CO_2_ (standardized to 25°C)	μmol m^−2^ s^−1^Pa^−1^	CiCc Models
*V_*cmax*_ (V_*cmax25*_)*	Maximum rate of carboxylation (standardized to 25°C)	μmol m^−2^ s^−1^	All
*J_*max*_ (J_*max25*_)*	Maximum rate of electron transport (standardized to 25°C)	μmol m^−2^ s^−1^	Jm models
ϕ_*J*_	Quantum yield estimate using Equation (3.6)	e^−^ photon^−1^	Jm models
θ_*J*_	Curvature factor photosynthetic light response curve	unitless	Jm models

*Equations described in Table 2. Model coding described in Table 3*.

**Table 2:** List of equations used in eight photosynthesis models.

**Equation No**.	**Equation**	**Description (models using equation)**
3.1	*A*_*exp*_ = *min*(*A*_*c*_, *A*_*j*_)	Expected CO_2_ assimilation rate as minimum of 2 limiting factors (All)
3.2	$\begin{array}{cc} A_{i}= & \frac{-b+\sqrt{b^{2}-4ac}}{2a} \end{array}$	General quadratic form for solving *A_c_*, *A_j_* (All)
3.3*a*	$\begin{array}{l} a=\frac{-1}{g_{m}}\\ b=\frac{V_{cmax}-R_{d}}{g_{m}}+C_{i}+K_{c}(\frac{1+O}{K_{o}})\\ c=R_{d}(C_{i}+K_{c}(\frac{1+O}{K_{o}}) \end{array}$	*A*_*c*_ solution using intercellular CO_2_(*C_i_*) and mesophyll conductance (*g_m_*) (CiCc_Jf, CiCc_Jm, CiCc_Jf_Temp, CiCc_Jm_Temp)
3.3*b*	$\begin{array}{l} a=\frac{-1}{g_{s}}\\ b=\frac{V_{cmax}-R_{d}}{g_{s}}+C_{a}+K_{c}(\frac{1+O}{K_{o}})\\ c=R_{d}(C_{a}+K_{c}(\frac{1+O}{K_{o}}) \end{array}$	*A*_*c*_ solution using ambient CO_2_(*C_a_*) and stomatal conductance (*g_s_*) (CaCc_Jf, CaCc_Jm, CaCc_Jf_Temp, CaCc_Jm_Temp)
3.4*a*	$\begin{array}{cc} a= & \frac{-1}{g_{m}}\\ b= & \frac{\frac{J_{i}}{4}-R_{d}}{g_{m}}+C_{i}+2\Gamma^{*}\\ c= & R_{d}\left(C_{i}+2\Gamma^{*}\right)-\frac{J_{i}}{4}\left(C_{i}-\Gamma^{*}\right) \end{array}$	*A*_*J*_ solution using *C_i_* and *g_m_* (CiCc_Jf, CiCc_Jm, CiCc_Jf_Temp, CiCc_Jm_Temp)
3.4*b*	$\begin{array}{cc} a= & \frac{-1}{g_{s}}\\ b= & \frac{\frac{J_{i}}{4}-R_{d}}{g_{s}}+C_{a}+2\Gamma^{*}\\ c= & R_{d}\left(C_{iobs}+2\Gamma^{*}\right)-\frac{J_{i}}{4}\left(C_{a}-\Gamma^{*}\right) \end{array}$	*A*_*J*_ solution using *C_a_* and *g_s_* (CaCc_Jf, CaCc_Jm, CaCc_Jf_Temp, CaCc_Jm_Temp)
3.5	$\begin{array}{cc} J_{f}= & \frac{F_{m}^{\prime }-F_{s}}{F_{m}^{\prime }}fQ\alpha_{leaf} \end{array}$	Chlorophyll fluorescence derivation of electron transport rate (ETR) (*J_f_*) (CiCc_Jf, CaCc_Jf, CiCc_Jf_Temp, CaCc_Jf_Temp)
3.6	*a* = θ_*J*_ *b* = −(Q*ϕ_J_*α_*leaf*_) − *Jmax* *c* = *Qϕ*_J_*Jmax	Quadratic roots for whole chain ETR (*J_m_*) (CiCc_Jm, CaCc_Jm, CaCc_Jm_Temp, CiCc_Jm_Temp)
3.7	$\begin{array}{cc} P= & P_{25}exp\left[\frac{E_{i}\left(T-298\right)}{298RT}\right] \end{array}$	Arrhenius temperature response for parameter (*P*) (CiCc_Jf_Temp, CiCc_Jm_Temp, CaCc_Jf_Temp, CaCc_Jm_Temp)

**Table 3:** Coding of eight photosynthesis models based on three contrasting assumptions with the total number of structural parameters in each model.

**Model**	**Temperature constraint on parameters**	***g_*m*_* limitation estimated**	**ETR derived using Equation (3.5)**	**ETR derived using Equation (3.6)**	**Number of structural parameters**
CiCc_Jf		X	X		6
CiCc_Jm		X		X	9
CaCc_Jf			X		5
CaCc_Jm				X	8
CiCc_Jf_Temp	X	X	X		12
CiCc_Jm_Temp	X	X		X	16
CaCc_Jf_Temp	X		X		10
CaCc_Jm_Temp	X			X	14

**Table 4:** Prior probability distributions of parameters used in eight photosynthesis models.

**Parameter**	**Prior distribution dnorm (mean, precision)**	**Prior type**	**Citation(s)**
*E_*gm*_*	Dnorm (49.6, 0.1)	Broadly informed (geno)	Ethier and Livingston, 2004; Sharkey et al., 2007; Patrick et al., 2009; Zhu et al., 2011
*E_*Jmax*_*	Dnorm (46.1, 0.01)	Broadly informed (geno)	Leuning, 2002; Medlyn et al., 2002a; Sharkey et al., 2007; Patrick et al., 2009; Zhu et al., 2011
*E_*Kc*_*	Dnorm (70.4, 0.5)	Broadly informed (geno)	von Caemmerer, 2000; Ethier and Livingston, 2004; Sharkey et al., 2007; Patrick et al., 2009; Zhu et al., 2011
*E_*Ko*_*	Dnorm (36.0, 0.5)	Broadly informed (geno)	von Caemmerer, 2000; Ethier and Livingston, 2004; Sharkey et al., 2007; Patrick et al., 2009; Zhu et al., 2011
*E_*Rd*_*	Dnorm (63.9, 0.1)	Broadly informed (geno)	Bernacchi et al., 2001; Ethier and Livingston, 2004; Sharkey et al., 2007; Zhu et al., 2011
*E_*Vcmax*_*	Dnorm (65.4, 0.5)	Broadly informed (geno)	Leuning, 2002; Medlyn et al., 2002a; Sharkey et al., 2007; Patrick et al., 2009; Zhu et al., 2011
$E_{\Gamma^{*}}$	Dnorm (26.8, 0.5)	Broadly informed (geno)	von Caemmerer, 2000; Ethier and Livingston, 2004; Sharkey et al., 2007; Patrick et al., 2009; Zhu et al., 2011
*g_*m(25)*_*	Dnorm (2.5, 0.025)	Broadly informed (ind)	Ethier and Livingston, 2004; Sharkey et al., 2007; Patrick et al., 2009; Zhu et al., 2011
*J_*max(25)*_*	Dnorm (171,0.000308)	Well-informed, C3 crops (ind)	Wullschleger, 1993
*K_*c(25)*_*	Dnorm (27.24, 0.5)	Broadly informed (geno)	von Caemmerer, 2000; Sharkey et al., 2007; Patrick et al., 2009; Zhu et al., 2011
*K_*o(25)*_*	Dnorm (30400,1.0 × 10^−5^)	Broadly informed (geno)	von Caemmerer, 2000; Sharkey et al., 2007; Patrick et al., 2009; Zhu et al., 2011
*R_*d(25)*_*	Dnorm (1.17, 1)	Broadly informed (ind)	Zhu et al., 2011
*V_*cmax(25)*_*	Dnorm (90, 0.000625)	Well-informed, C3 crops (ind)	Wullschleger, 1993
$\Gamma_{\left(25\right)}^{*}$	Dnorm (3.86,10)	Broadly informed (ind)	von Caemmerer, 2000; Sharkey et al., 2007; Patrick et al., 2009; Zhu et al., 2011
θ_*J*_	Dnorm (0.8, 10)	Broadly informed (ind)	Lambers et al., 2008
ϕ_*j*_	Dnorm (0.4, 10)	Broadly informed (ind)	Lambers et al., 2008

*Descriptions of parameters are given in Table 1. Prior distributions are based on summaries of literature listed in citations. Distributions are described as broadly informed from data on C3 species, well-informed from data on C3 crops. Prior type indicates the traits hierarchical level as genotypic estimation only (geno) or genotype and individual plant level (ind). Prior distributions for individual level traits describe genotype level mean*.

All models assumed that observations of *A* (*A*_*n*_) (μmol m^−2^ s^−1^) followed a normal distribution:

(1) $$A_{n}\;\sim \;N(A_{exp},\tau )$$

where *A*_*exp*_ is the expected photosynthetic rate, τ is precision (1/σ^2^) describing the variability in measurement error. A hierarchical design nested individual plant parameters within the genotypic populations. This nested design was used for all eight photosynthesis models with each model and genotype run independently. Parameters undergoing individual and genotypic estimation employed normal distributions following:

(2) $$\mu Y_{i}\;\sim \;N\left(\mu Y_{geno},\tau_{\text{Ygeno}}\right)$$

where μ*Y*_*i*_ is individual level parameters means, μ*Y*_*geno*_ are genotypic parameters means and τ_Ygeno_ is the genotypic precision (1/σ^2^). Table 4 shows the parameters estimated at both the individual and genotypic level as well as those only estimated genotypically. The choice of parameters estimated at genotypic level considered both evolutionary constraints for *K*_*c*_ and *K*_*o*_ (Galmes et al., 2005) and an analysis of trait variance using a suite of non- hierarchical models for *E*_*i*′_s (data not shown). The variance priors for individual level parameters used weakly informed gamma distributions. A weakly informed Folded-Cauchy distribution (implemented as a truncated t-distribution with one degree of freedom) was used to describe prior distribution for process model variance structure (τ) (Gelman, 2006). This was centered at 0, set at the range of [0,∞) with a standard deviation of 2.5.

### Photosynthesis models

Within plant physiology and earth system science the Farquhar, von Caemmerer and Berry model of photosynthesis (FM) stands out for its mechanistic, principally biophysical/biochemical, basis for modeling C3 photosynthesis (Farquhar et al., 1980; von Caemmerer, 2000). FM, developed at the leaf scale, originally proposed two rate limiting factors controlling *A* by finding the minimum of RuBisCO limited *A, A*_*c*_, and RuBP regeneration limited *A, A*_*J*_ (Farquhar et al., 1980), with a *g*_*m*_ limitation added subsequently (Ethier and Livingston, 2004). A triose phosphate utilization limitation (*TPU*) of *A* (Sharkey et al., 1985) was considered using a similar quadratic structure. Results showed *TPU* affecting *A* at greater than 93.6 μmol m^−2^ s^−1^, above the maximum *A*_*n*_ (70.3 μmol m^−2^ s^−1^) found in our data, and therefore a *TPU* limitation was not included.

Modeling equations are presented in Table 2, and all approaches used the general quadratic form of FM, Equation (3.2). All models predicted both *A*_*c*_ and *A*_*J*_ but varied in their inclusion of three components: (1) the use of a temperature constraint on model parameters, (2) the inclusion or absence of a *g*_*m*_ limitation, and (3) the derivation for estimating *A*_*J*._ This 2^3^ design leads to eight modeling formulations when accounting for all combinations. First a subset of models included a temperature constraint on parameters, others differed in the inclusion or absence (assuming infinite) of a *g*_*m*_ limitation (Equations 3.3 a or b and 3.4 a or b), and finally a subset used two alternative characterizations of ETR, following either Equation (3.5) or (3.6) (Table 2). Models were developed on all combinations of these three assumptions (Table 3). Models with a *g*_*m*_ limitation were coded CiCc, while infinite *g*_*m*_ models were coded CaCc. Models using Equation (3.5), fluorescence derived ETR, were coded with a Jf, and models using Equation (3.6) to derive ETR were coded with a Jm. Models with a temperature constraint on parameters had an added Temp in model identifier.

Mesophyll conductance has been demonstrated to limit *A* and has been integrated into the FM (Ethier and Livingston, 2004; Flexas et al., 2008). Increasingly, *g*_*m*_ is being shown to impact photosynthesis under stressful conditions (Flexas et al., 2008; Niinemets et al., 2009b; Tomás et al., 2014). It is, however, still common practice to assume an infinite *g*_*m*_ (Thornton et al., 2005; Kattge et al., 2009) due to limited knowledge of its interspecific variation and dynamics as well as the challenges and costs associated with some estimation techniques (Niinemets et al., 2009a; Gu and Sun, 2014; Hanson et al., 2016). Here we integrated a *g*_*m*_ limitation in four models using a curve fitting approach following Equations (3.3a) and (3.4a) (Pons et al., 2009). In a subset of these *g*_*m*_ itself was given a temperature dependency (Bernacchi et al., 2002).

Chlorophyll fluorescence measurements are widely used in plant physiological investigations, including for the quantification of photosystem II (PSII) operating efficiency and fluorescence derived ϕ_*PSII*_ (Genty et al., 1989; Maxwell and Johnson, 2000). There remains considerable uncertainty in using the fluorescence derivation of ETR (Equation 3.5) for describing *A*_*J*_ (Maxwell and Johnson, 2000; Baker, 2008). Here we tested the utility of fluorometry calculated ETR based on two assumptions. First, leaf absorptance (α_*leaf*_), assuming α_*blue*_ and α_*red*_ of 0.92 and 0.87 respectively, was used to establish absorbed photosynthetically active photon flux density (*Q*) (He et al., 2007). Second, the partitioning of energy between photosystem I (PSI) and PSII (*f)* was assumed equal at 0.5. Because of the limitations in using fluorometry derived ETR when no alternate electron routes are included, we also considered a classically derived empirical model to estimate ETR, following Equation (3.6) (von Caemmerer, 2000). This formulation required the parameterization of *J*_*max*_, ϕ_*J*_ and a light response curvature parameter (θ_*J*_).

Temperature dependencies have been developed and demonstrated for RuBisCO activity, mediated by the Michaelis-Menten enzymatic constants *K*_*c*_ and *K*_*o*_, as well as *V*_*cmax*_, *R*_*d*_, Γ^*^, g_*m*_, and *J*_*max*_ (Bernacchi et al., 2002; Medlyn et al., 2002a). For models that assume a temperature constraint, we used an Arrhenius style temperature response function, Equation (3.7) (Bernacchi et al., 2002; Medlyn et al., 2002b; Patrick et al., 2009). This simple model required the estimation of one temperature response parameter (*E*_*i*_) representing the activation energy. Estimates of temperature dependency were made for six parameters in total (*V*_*cmax*_, *J*_*max*_, *R*_*d*_, Γ^*^, g_*m*_, *K*_*c*_, *and K*_*o*_) with *E*_*Vcmax*_, *E*_*Rd*_, $F_{\Gamma^{*}}$, *E*_*Kc*_, *and E*_*Ko*_ estimated in all Temp models, *E*_*Jmax*_, estimated in Jm_Temp models, and *E*_*gm*_ estimated CiCc_Temp models.

### Model computation

We used Gibbs Sampling, a Markov Chain Monte Carlo (MCMC) method, to generate the posterior distributions of parameters (θ) and errors (Gelman and Rubin, 1992; Kruschke, 2010). Sampling was conducted with rjags within the R Foundation for Statistical Computing (Plummer, 2014; R Development Core Team, 2014). After a burn in period of 200,000 iterations, four independent MCMC chains were run for 250,000 iterations for each model by genotype. Each chain was sampled every 20th frame yielding 50,000 samples per model per genotype. Across models, a univariate potential scale reduction factor ($\sqrt{\hat{R}}$) provided a convergence diagnostic for each parameter. A multivariate potential scale reduction factor ($\sqrt{{\hat{R}}_{M}}$) provided a single convergence metric for the entire model (Brooks and Gelman, 1998). In all we evaluated eight alternative model structures on 31 individuals from six genotypes of *B. rapa* resulting in 248 unique parameterizations of *A/Ci* response. Diagnostics of the four-chain convergence were conducted using visual inspection of trace and density plots demonstrating chain convergence in similar sample space. Chain convergence diagnostic tools $\sqrt{\hat{R}}$ and $\sqrt{{\hat{R}}_{M}}$ did not exceed the recommended maximum of 1.2, with maximums across all individuals and models of 1.06 and 1.01 for the univariate and multivariate convergence statistics respectively.

### Model scoring metric

To quantitatively compare model results for each individual, genotype, and species, we used the Deviance Information Criterion (DIC), a Bayesian analog to Akaike Information Criterion (Spiegelhalter et al., 2002). DIC considers all models to be conceptually equal and acts purely as a model scoring metric not an analytical evaluation of model functional form (Gelman et al., 2004). DIC is calculated by combining a deviance term and a complexity penalty term (Spiegelhalter et al., 2002). The Bayesian model deviance (*D*(θ)) is based on the residuals between the model and the data, computed with

(3) D(θ)= −2log[p(Y |θ)]+2log[f(Y)]

where *Y* is observed data, θ represents all parameters for the model, *p*(*Y*|θ) is the likelihood function defined by the model, and *f*(*Y*) is a standardizing term remaining constant for all models and therefore having no influence model comparison. The Bayesian deviance alone is not a strong model discrimination metric as higher dimensional models could be favorably biased. DIC attempts to account for this bias with a parameterization penalty (Spiegelhalter et al., 2002; Plummer, 2008). The penalty, plug-in deviance (*pD*), from the Spiegelhalter derivation is $pD=\bar{D\left(\theta \right)}-D\bar{\left(\theta \right)}$, where *D*(θ) is posterior mean of the deviance using all parameters samples of sequence and *D*(θ) is deviance evaluated at the posterior mean of the all parameters. In the calculation of DIC the posterior distribution of *D*(θ) is used to express mean deviance following

(4) $$DIC=\;D\bar{\left(\theta \right)}+2pD.$$

$D\bar{\left(\theta \right)}$ and $\bar{D\left(\theta \right)}$ are easily calculated from MCMC output through the monitoring of *D*(θ) of all simulated values. ΔDIC is calculated as the difference between model DIC score and the genotype minimum DIC score. No significant ΔDIC has been universally accepted, however differences of ten are often employed (Spiegelhalter et al., 2003) and used here.

### Parameter variability

We evaluated full model sets of trait posteriors through the development of multiple posterior predictive checks; using the posterior trait distributions to simulate *A, A*_*exp*_, while considering the uncertainty in posterior distributions (Kruschke, 2013). Two methods were then used to compare posterior trait distributions. First, boxplots of posterior parameter distributions were compared across genotypes and against the prior probability distributions. Second, high-density intervals (HDIs) were used at eight percentiles (50, 60, 70, 80, 85, 90, 95, and 99%). HDI is a Bayesian posterior comparison metric identifying portions of the posterior distributions having a higher probability density than regions outside that interval (Kruschke, 2010). To describe the relative credibility of trait variance the differences in the posterior mean distributions were taken for all traits within a given model (Kruschke, 2013). This difference was then evaluated for intersection with zero at the eight percentiles listed above. The maximum HDI percentile of the posterior trait differences, which did not intersect with zero, was used to describe degree of credible trait variance between two genotypes. This HDI differencing test was conducted across all genotypes and traits within each model.

### Model sensitivity analysis

To evaluate consistency in model performance a sensitivity analysis was conducted. The entire analysis was rerun after adding Gaussian noise with a mean of 0.0 and standard deviation of 2.0 μmol m^−2^ s^−1^ to the *A*_*n*_ data. This was chosen to mimic instrumentation error of IRGA observations. All statistical analysis was conducted in the in R software environment (R Development Core Team, 2014).

## Results

### Model performance

**Figure 2:**
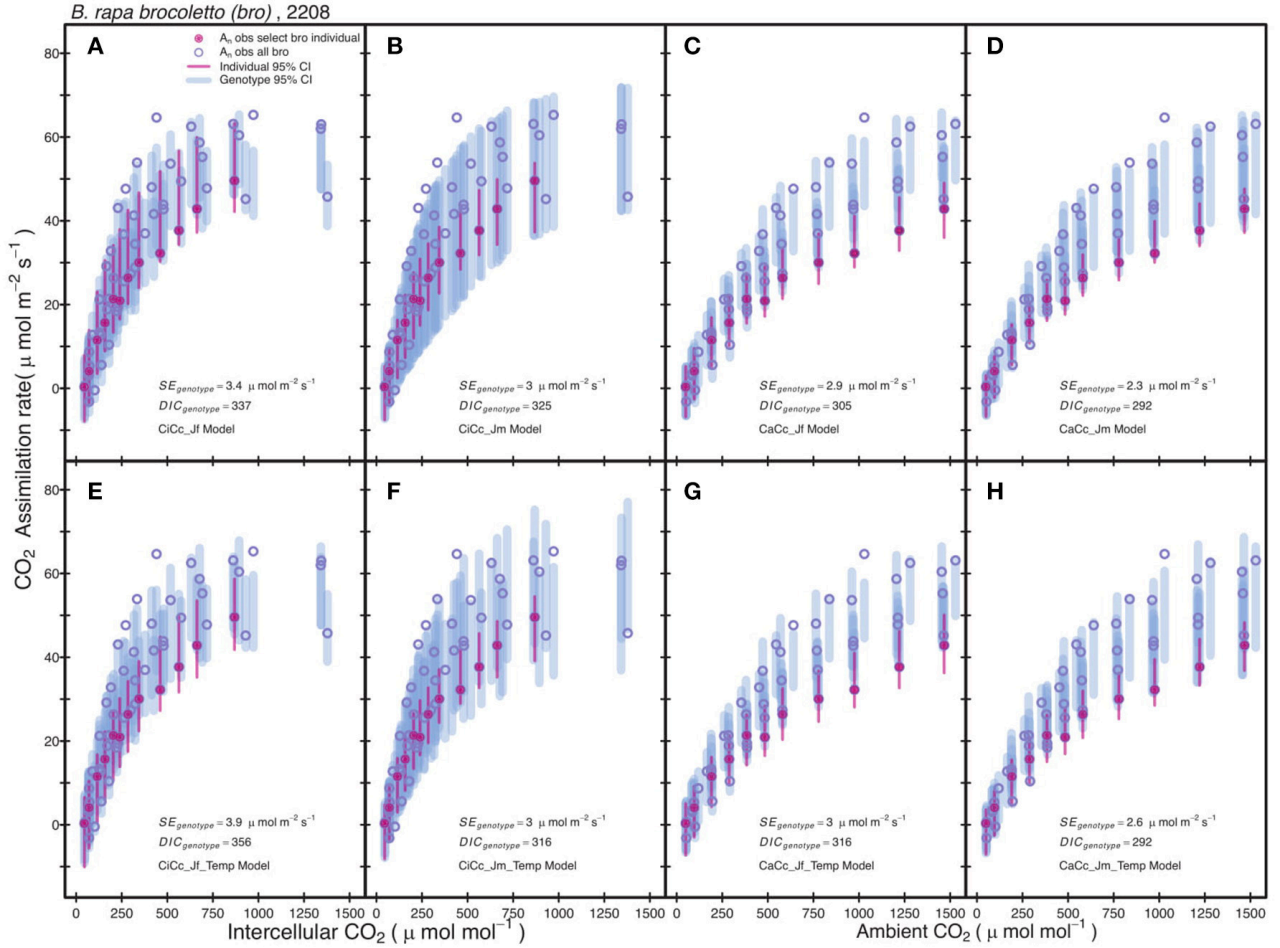
Comparison between observed CO_2_ assimilation (*A*_*n*_) and estimated assimilation for eight photosynthesis models of *B. rapa*, var. *bro*, including a single individual level estimate. Each plot shows *A*_*n*_ for a select *bro* individual (filled-circle) and *A*_*n*_ for all other *bro* data (open-circle). The Bayesian 95% credible intervals (vertical lines) are shown at individual level (thin-line) and genotypic (thick-line). Models differ in three assumptions: **(A–D)** have no temperature constraint on parameters while **(E–H)** use an Arrhenius style temperature constraint on model parameters, **(A,C,E,G)** use an estimate of ETR derived from Equation (3.5) based on chlorophyll fluorescence, **(B,D,F,H)** estimate ETR from Equation (3.6), **(A,B,E,F)** predict mesophyll conductance limitations using intercellular CO_2_ observations while **(C,D,G,H)** assume infinite mesophyll conductance using ambient CO_2_ observations.

Posterior parameter distributions were used to predict *A*_*c*_ and *A*_*J*_ for each model to compute *A*_*exp*_ at individual and genotypic levels. The genotype level mean standard error of *A*_*exp*_ for all models was 2.75 μmol m^−2^ s^−1^ with a minimum of 0.9 μmol m^−2^ s^−1^ for *r46* in the CiCc_Jm model and a maximum of 7.29 μmol m^−2^ s^−1^ for *tur* in the CiCc_Temp_Jf model. The *A/Ci* observations (*A*_*n*_ vs. *C*_*i*_ or *C*_*a*_) of the modeled data are shown along with 95% genotypic credible intervals (CI) for *bro* along with 95% individual CI for selected *bro* individual (Figure 2). Individual level CI's fell mostly within genotypic CI in Figure 2, with the exception of CaCc Jf models where individual CI's fell below genotypic at high CO_2_ availability.

**Figure 3:**
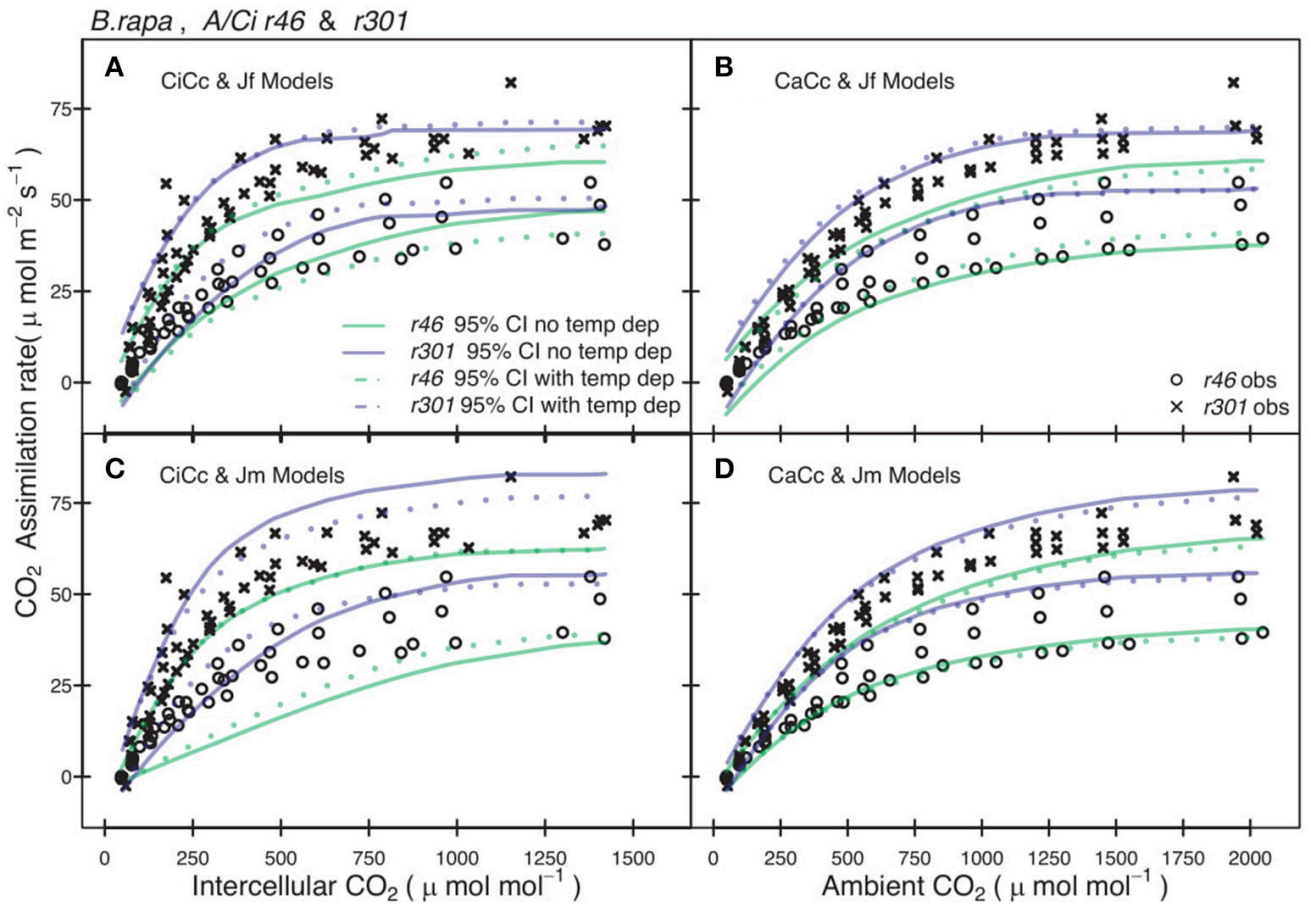
Comparison between observed CO_2_ assimilation (*A*_*n*_) and predicted 95% credible interval (CI) for eight models of two *B. rapa* genotypes (*r46, r301*). Each plot shows *A*_*n*_ for both *r46* (open circles) and *r301* (closed circles) and Bayesian 95% CI for two models one with temperature constraint (dotted lines) the other without (solid lines), using genotype level posterior trait distributions. **(A)** Models shown incorporated an estimate of mesophyll conductance (*g*_*m*_) and used chlorophyll fluorescence (Equation 3.5, Table 2) to characterize electron transport rate (ETR). **(B)** Models assumed an infinite *g*_*m*_ and used Equation (3.5) to characterize ETR. **(C)** Models shown incorporated an estimate of *g*_*m*_ and used Equation (3.6) to characterize ETR. **(D)** Models assumed an infinite *g*_*m*_ and used Equation (3.6) to characterize ETR. Smoothed CI's using Loess fit to present multiple models together.

**Figure 4:**
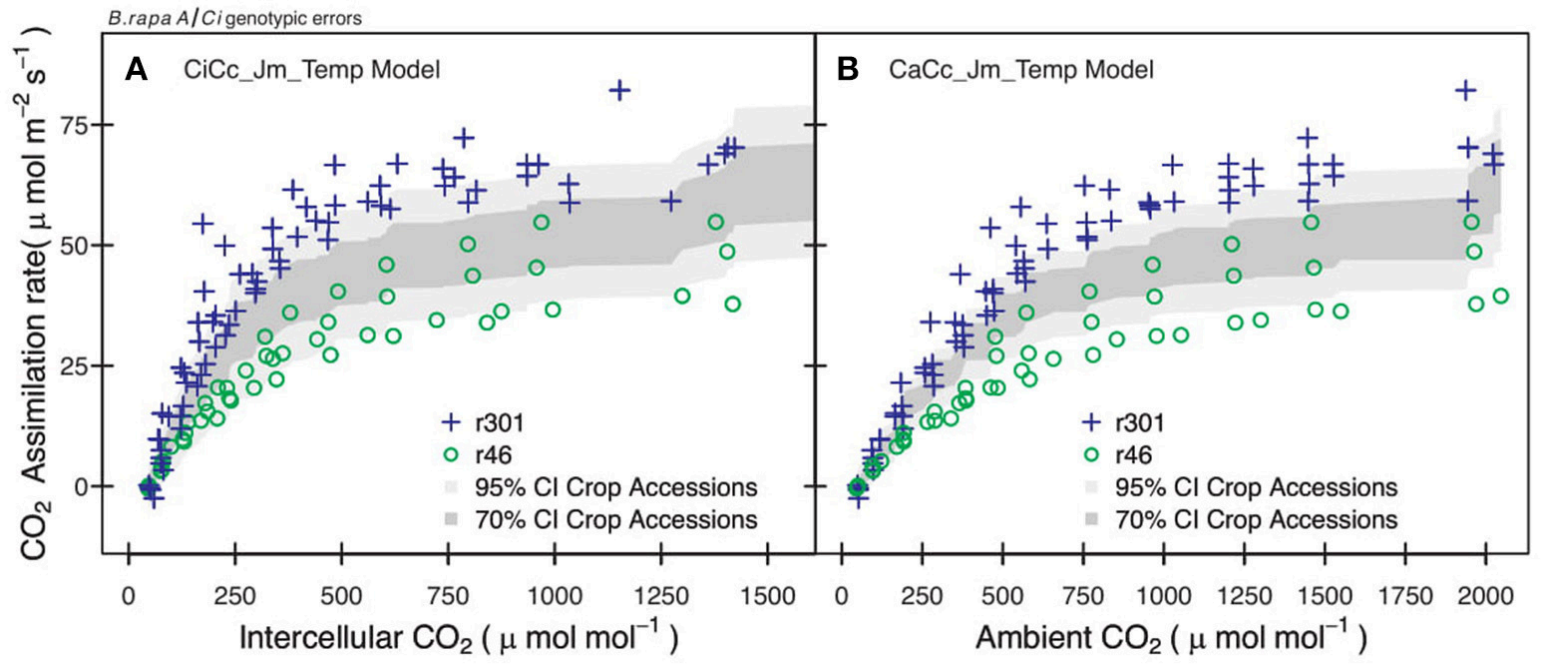
Examples of errors from non-genotypic parameterization for two *B.rapa* genotypes. **(A)** Observation points of *r46* (open circles) and *r301* (plus) with 95% (light gray) and 70% (dark gray) credible interval for CiCc_Jm_Temp model using combined parameter distributions from agricultural accessions (*bro, cab, oil, tur*). **(B)** Observation points of *r46* and *r301* with 95% and 70% credible intervals for CaCc_Jm_ Temp model using combined posterior parameter distributions of accessions.

Genotypic posterior trait distributions were used to construct 95% CIs on *A*_*exp*_. Figure 3 shows the *A/Ci* observations (*A*_*n*_ vs. *C*_*i*_ or *C*_*a*_) for all *r46* and *r301* individuals with the 95% CI for each model. A narrower range in 95% CIs was found in models assuming infinite *g*_*m*_ for *r46* and *r301* (Figure 3) as well as crop accessions (data not shown). Models estimating ETR using fluorescence (Jf models) showed lower overall 95% CIs on *A* than Jm models in all genotypes except *r46*. This is seen in the larger number of points beyond the upper CI limit for *r301* in Figures 3A,B; this same result was also found across crop accessions (data not shown). Finally, to evaluate genotypic vs. species level parameterization, an accession level parameterization was developed and used to predict the RILs *A/C*_*i*_ response (Figure 4). Data from both RILs, most noticeably *r301*, fell outside the 95% accession based CI (Figure 4).

### Model structural comparison

Genotypic model DIC scores were used to compute ΔDIC along with genotype pD's (Table 5). For the species, the CaCc_Jm_Temp and the CaCc_Jm models were top tier models for four of the six genotypes, with the exceptions being *cab* and *r301*. CiCc_Jm, CaCc_Jf, and CaCc_Jf_Temp were each included in one genotypes' top-tier, *r301, oil* and *oil*, respectively. CiCc_Jf, CiCc_Jf_Temp, and CiCc_Jm_Temp failed to have a ΔDIC of less than 10 across genotypes. The pD's were consistently highest in models deriving ETR using Eqn 3.6 relative to fluorescence derived ETR (Equation 3.5). pD's were also consistently higher in models assuming a *g*_*m*_ limitation relative to infinite *g*_*m*_.

**Table 5:** Genotype DIC increment with respect to genotype minimum (ΔDIC) with mean effective number of parameters (pD) for eight models.

	**Genotype ΔDIC (pD)**					
**Model**	***r301***	***r46***	***bro***	***cab***	***oil***	***tur***
CiCc_Jf	165.0 (11.5)	129.6 (9.7)	45.1 (13.4)	115.6 (12.0)	50.5 (5.7)	113.4 (8.9)
CiCc_Jm	**0** (20.8)	68.4 (28.7)	32.9 (12.7)	17.8 (16.0)	27.3 (8.9)	21.3 (53.0)
CaCc_Jf	138.5 (9.1)	85.1 (6.5)	13.2 (5.4)	81.1 (5.2)	**0** (7.9)	96.8 (8.1)
CaCc_Jm	68.1 (16.4)	**0** (12.4)	**0** (12.9)	**0** (9.0)	23.3 (11.5)	31.4 (20.1)
CiCc_Jf_Temp	210.2 (7.0)	129.1 (5.6)	64.5 (11.5)	156.0 (8.5)	45.2 (10.7)	182.7 (6.5)
CiCc_Jm_Temp	17.6 (17.0)	54.8 (15.8)	24.4 (11.4)	39.1 (29.3)	21.2 (13.4)	15.1 (24)
CaCc_Jf_Temp	134.9 (10.1)	98.7 (5.5)	17.0 (8.2)	81.3 (12.9)	**5.8** (9.4)	70.0 (6.3)
CaCc_Jm_Temp	60.4 (16.6)	**9.9** (13.8)	**0.6** (8.8)	15.3 (19)	18.3 (7.5)	**0** (14.3)

*Bolded are models with top-tier ΔDIC scores for respective genotypes using ΔDIC threshold of 10*.

### Parameter differentiation

**Figure 5:**
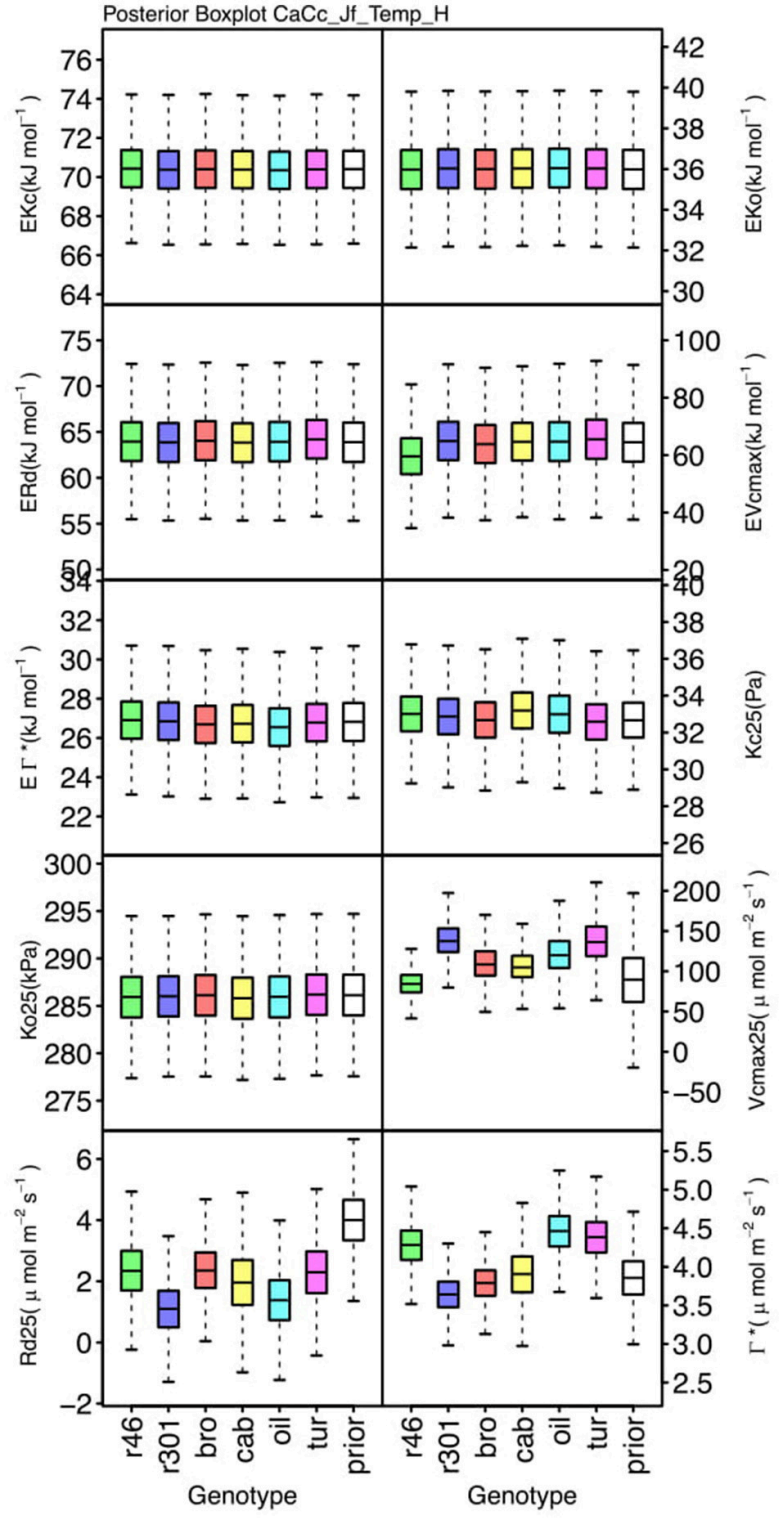
Boxplots (median, quartiles, minimum, maximum) of posterior distributions of all parameters in CaCc_Jf_Temp model, which are described in Table 1 and priors for each parameter (Table 4).

**Figure 6:**
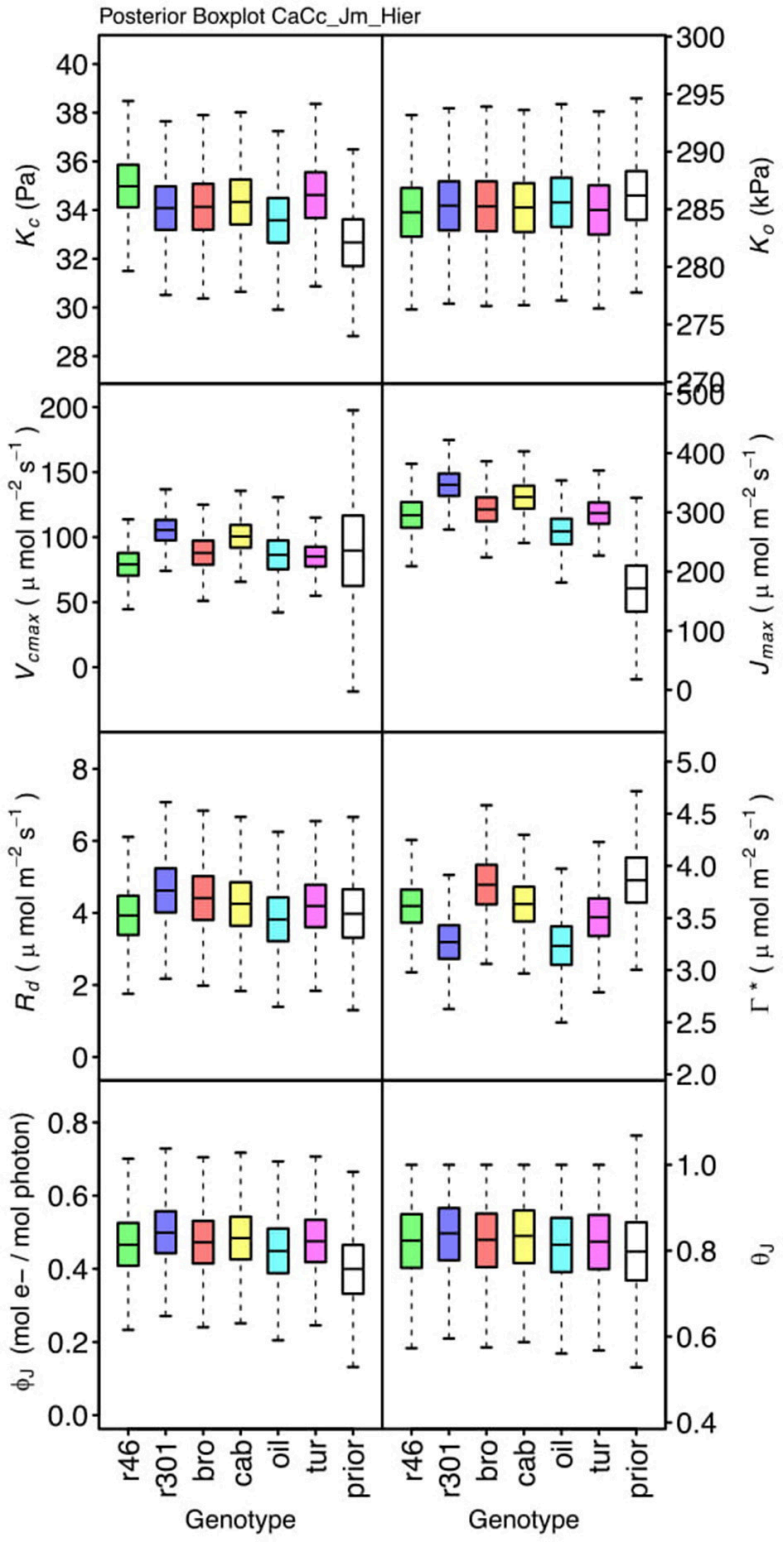
Boxplots (median, quartiles, minimum, maximum) of posterior distributions of all parameters in CaCc_Jm model, which are described in Table 1 and priors for each parameter (Table 4).

**Figure 7:**
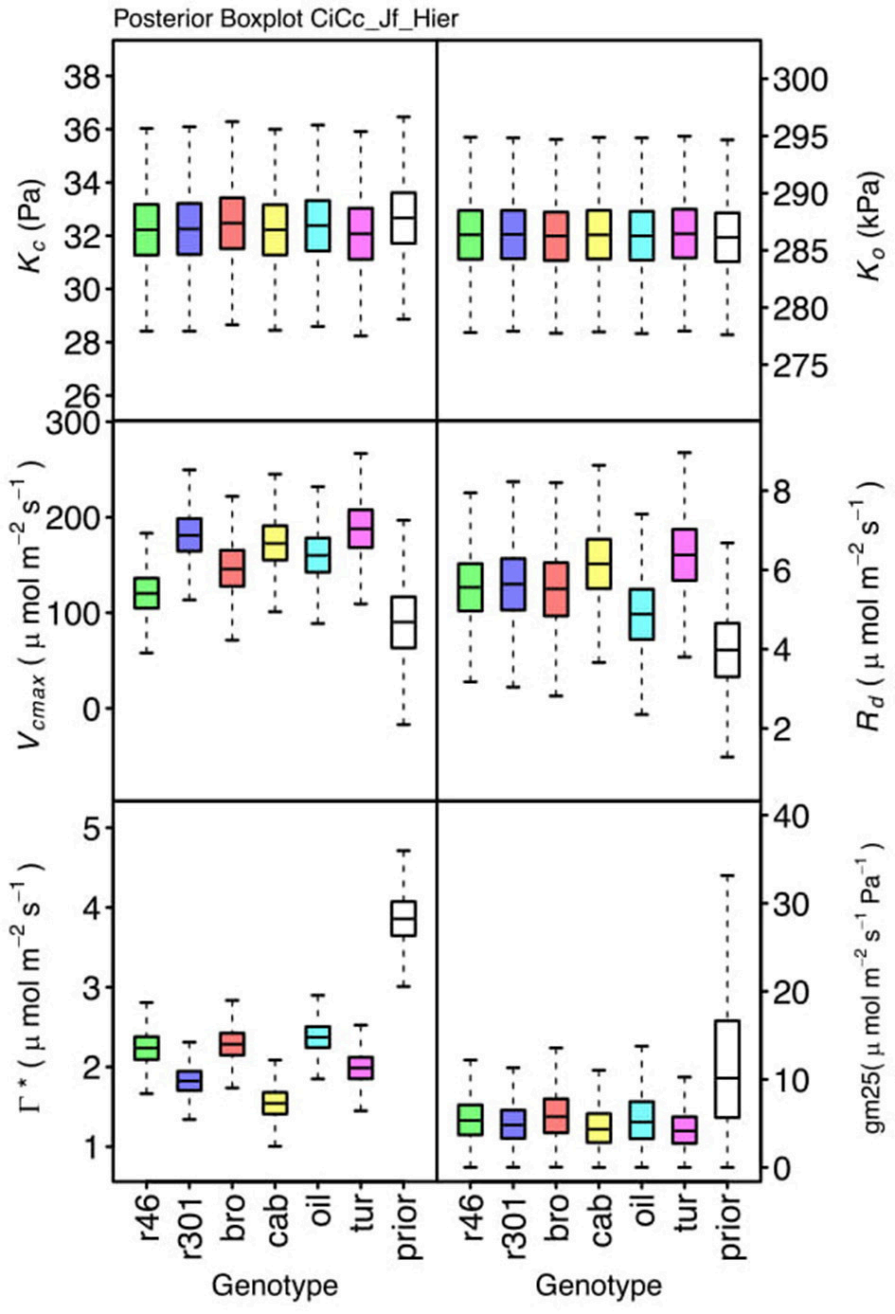
Boxplots (median, quartiles, minimum, maximum) of posterior distributions of all parameters in CiCc_Jf model, which are described in Table 1 and priors for each parameter (Table 4).

**Figure 8:**
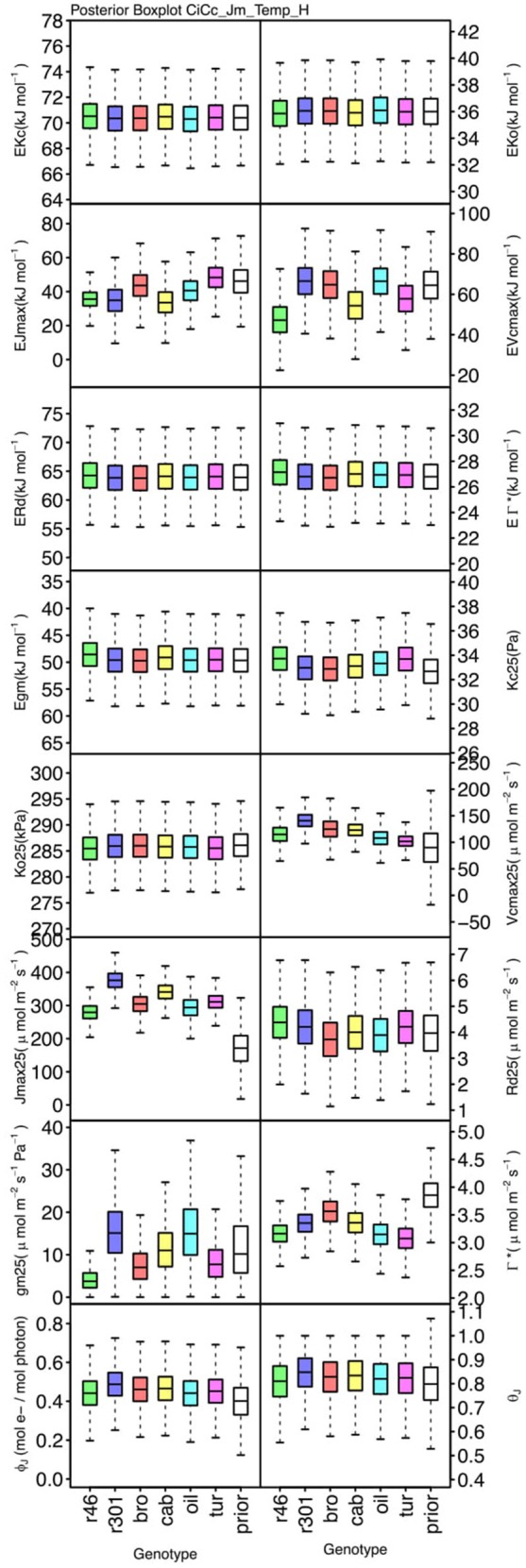
Boxplots (median, quartiles, minimum, maximum) of posterior distributions of all parameters in CiCc_Jm_Temp model, which are described in Table 1 and priors for each parameter (Table 4).

Genotypic posterior trait distributions were compared using posterior boxplots and an analysis of HDI's. Trait distributions showed some parameters with high probability of genotypic variation, including *J*_*max*_, *V*_*cmax*_, Γ^*^, and *E*_*Vcmax*_, and traits with limited probability of variation, including *K*_*o*_, ϕ_*J*_ and θ_*J*_ (Figures 5–8). *V*_*cmax*_, Γ^*^, R_*d*_, *K*_*c*_, and *K*_*o*_ were the five traits estimated in all eight models. *V*_*cmax*_ showed genotypic variance in all models with *r46* notably lower than other genotypes in all Jf models (Figures 5, 7). Γ^*^ also showed genotypic variance across models with lower estimates in *g*_*m*_ limited models (Figures 7, 8) than infinite *g*_*m*_ models (Figures 5, 6). *R*_*d*_ showed genotypic variance in five of the eight models, most pronounced in models CaCc_Jf and CaCc_Jf _Temp (Figure 5), while the only variance seen in *K*_*c*_ was in the CaCc_Jm model, with *r46* differing from *oil* (Figure 6). The temperature activation energies (*E*i's) showed limited probability of genotypic variance with two exceptions. *E*_*Vcmax*_ for *r46* was lower relative to other genotypes in the CiCc_Jf_Temp model and estimates in *r46* and *cab* were also lower in the CiCc_Jm_Temp models (Figure 8) *E*_*Jmax*_ also showed variance in models CiCc_Jf_Temp and CiCc_Jm_Temp (Figure 8). Amongst the ETR traits modeled using Equation (3.6) only *J*_*max*_ showed genotypic variation, this was found across all Jm based models (Figures 6, 8), the variation was dominated by higher estimates for *r301*.

**Figure 9:**
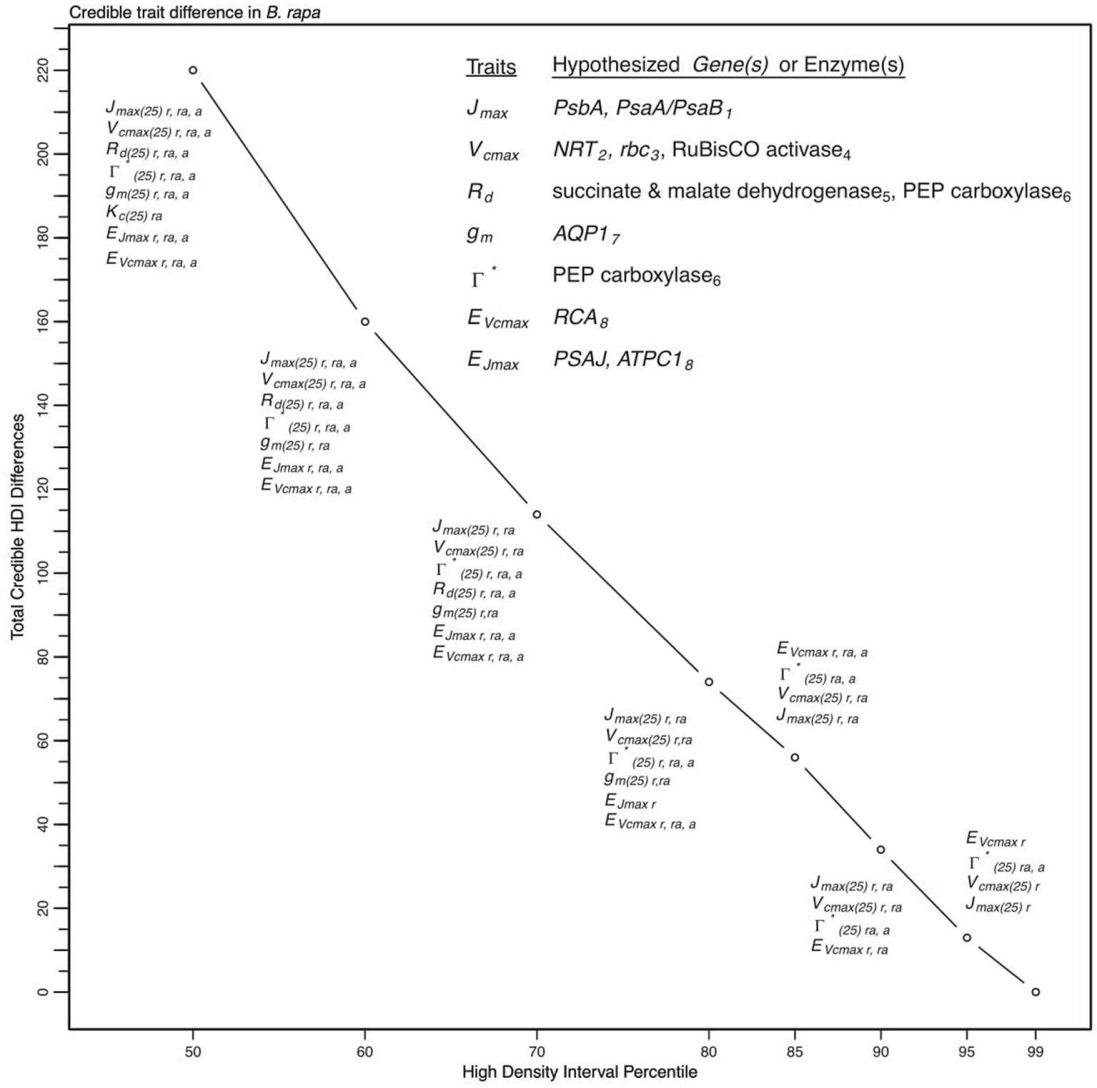
Illustration of trait potential for variation in represented *B. rapa* population along with prospective mechanistic underpinnings. The total number of instances where HDI percentile difference for a trait did not intersect with zero across eight models at eight HDI percentiles. At each percentile, the parameters identified with credible interval differences are listed as well as indication if the difference was between RILs (*r46* and *r301*) (r), between RILs and an agricultural accession (ra) or between two agricultural accessions (a). Zero credible interval differences were observed at 99% HDI. References for hypothesized genes (italics) and enzymes in subscript as follows: 1, Foyer et al. (2012); 2, Masclaux-Daubresse et al. (2010); 3, Hauser et al. (2015); 4, Yamori et al. (2012); 5, Araujo et al. (2012); 6, Häusler et al. (1999); 7, Hanba et al. (2004); and 8, Song et al. (2014).

To describe the magnitude of genotypic trait variance, the differences in posterior parameter distributions among genotypes were computed for each model. These differences were then evaluated at eight HDI percentiles for overlap with zero; the maximum HDI interval not overlapping with zero was selected as the probability of variance. *J*_*max*_, *V*_*cmax*_, Γ^*^, and *E*_*Vcmax*_ were found with a probability of variance at 95% HDI (Figure 9). At 80% *E*_*Jcmax*_ and *g*_*m*_ show differences, at 70% *R*_*d*_ showed differences and at 50% HDI *K*_*c*_ emerges as variable (Figure 9). At 50% HDI half of the 16 traits estimated showed variance. To summarize the posterior trait distributions across models Table 6 lists maximum HDI percentile of traits differences. Of note in Table 6, *J*_*max*_ is classified as highly variable, differences found at < 90% HDI, in all Jm based models. The variability in *J*_*max*_ is dominated by the contrast between the two RILS, with *r46*'s median posterior between 50-100 μmol m^−2^ s^−1^ < *r301*'s (Figures 6, 8). Variance in *V*_*cmax*_ is dominated by differences in *r46* relative to other genotypes; most notable in the CiCc_Jf, CaCc_Jf models (Figures 5, 7).

**Table 6:** Summary of genotypic trait variability using differencing of high density intervals (HDI).

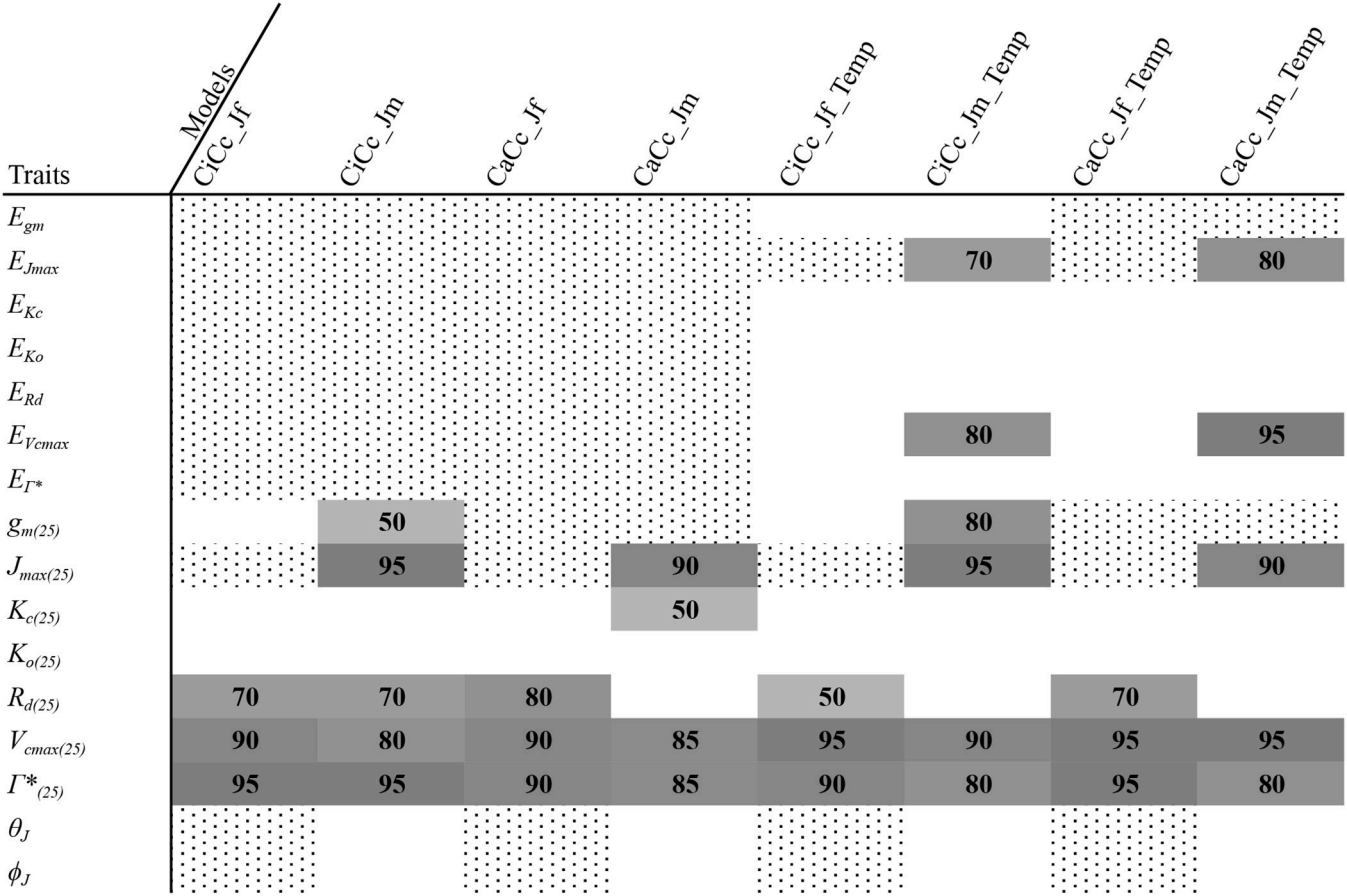

*Dot-pattern indicates traits not in a given model. White indicated no emergence of HDI difference at 50% HDI. Number indicates at which HDI percentile did the difference in posterior distributions no longer overlap with zero. Color coding from dark for largest HDI percentile (95) to light for smallest (50)*.

### Sensitivity analysis results

**Figure 10:**
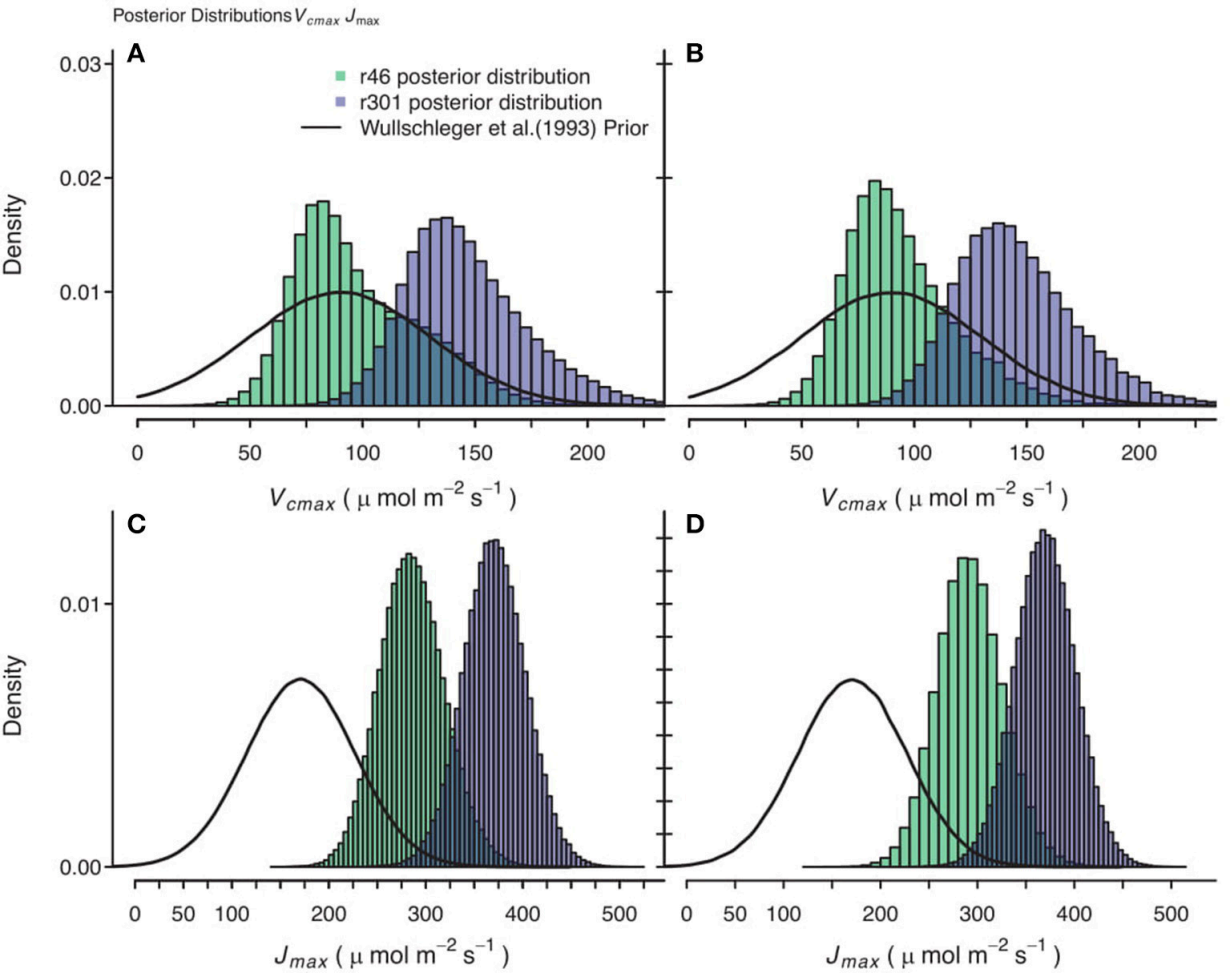
Comparison of posterior parameter distributions of two *B. rapa* genotypes (*r46, r301*) for two photosynthetic traits, maximum rate of carboxylation (*V*_*cmax*_) and maximum rate of electron transport Umax). **(A,C)** Show combined posterior distributions of all models for *V*_*cmax*_ and *J*_*max*_ respectively using observational data and prior on parameters based on Wullschleger (1993) for C3 crops. **(B,D)** Show combined posterior distributions of all individuals and models for *V*_*cmax*_ and *J*_*max*_, respectively using observational data with 2.0 μmol m^−2^ s^−1^ random noise added to assimilation data and using same Wullschleger (1993) prior.

Gaussian noise with mean of 0.0 μmol m^−2^ s^−1^ and standard deviation of 2.0 μmol m^−2^ s^−1^ was added to the *A*_*n*_ data, followed by a re-analysis. The resultant posterior parameter distributions were wider in some cases and some shifts in median estimates were seen, but no systematic trends were identified in these shifts. For example, in traits that play critical roles in the *A/C*_*i*_ response, the *J*_*max*_ noisy genotypic level median estimate was 2.2 μmol m^−2^ s^−1^ greater compared to the original analysis and for *V*_*cmax*_ the noisy median estimate was 4.4 μmol m^−2^ s^−1^ less than original analysis in the CiCc_Jm_Temp model. This is illustrated in comparing Figures 10A,C with Figures 10B,C.

## Discussion

### Multimodel approach

We show here that a multimodel based approach improves phenotypic information discovery in three critical ways. First, our trait analysis using a set of models identified potential genotypic differences requiring further investigation and revealed model components needing reevaluation (Table 5). Second, the Bayesian parameterization scheme revealed an expected trait hierarchy (Table 6). Finally, the multimodel approach provided greater confidence in estimates of trait variation among genotypes (Figure 9).

### Performance of models based on assumptions

The complexity analysis assessed the influence of factors not addressed experimentally (i.e., temperature) and of physiological mechanisms (i.e., *g*_*m*_ and ETR derivation) not yet characterized in the population under study. While a single preferred model structure was not identified using ΔDIC, we were able to evaluate the relative performance of model assumptions employed. First, DIC strongly favored derivation of ETR, and therefore *A*_*J*_, from Equation (3.6) (Table 5), as Jm based models were in the top tier in seven cases, while Jf models were rated as top-tier only two times and only for *oil*. This confirms previously identified limitations and illustrates the need to consider alternate e^−^ paths when using fluorometry to characterize *A*_*J*_ (Baker, 2008; Yin et al., 2009). Fluorometry estimates all PSII e^−^ excitation at the beginning of the e^−^ transport chain; using this to estimate the assimilatory outcome of e^−^ transport does not distinguish e^−^'s used for photosynthetic linear electron flow and the alternative pathways of e^−^ transport (Miyake, 2010). The biological relevance of these alternate pathways lies in the reduction of photooxidative stress (Foyer and Shigeoka, 2011), specifically the protection of PSII from heat and light stress (Miyake, 2010). Interestingly *oil* showed a preference for CaCc_Jf based models (Table 5). The divergence of *oil* from the other genotypes may be due to diminished flow to alternate pathways or a unique *f*. The parameter differentiation of *oil* reflects the allelic composition of that genotype, and warrants further investigation. An expanded genotypic sample may enable model modification for investigating alternate e- flow and *f* (Laisk and Loreto, 1996; Yin et al., 2009; Livingston et al., 2010).

Second, the combination of chlorophyll florescence derived ETR and *g*_*m*_-limitation (CiCc_Jf and CiCc_Jf_Temp) was not selected as a top tier model by any of the genotypes (Table 5), this shows the overall preference for both Equation (3.6) derivation of ETR and infinite *g*_*m*_. Interestingly, the preference for infinite *g*_*m*_ models based on ΔDIC emerged even though *g*_*m*_ as a trait was shown to vary in this population (Figure 8 and Table 6). A debate persists on the response of *g*_*m*_ to environmental conditions (Flexas et al., 2007; Tazoe et al., 2011) with possible mechanisms governing *g*_*m*_ behavior including anatomical components, biochemical changes such as aquaporin expression, and chloroplast surface area adjustment (Flexas et al., 2006; Chaumont and Tyerman, 2014; Tomás et al., 2014). Each of these may be variable within plant populations, and while both limited and ∞ *g*_*m*_ were viewed favorably here, further modeling work should aim for integration of *g*_*m*_ limitation, particularly in plants under stress and in those with intrinsically low *g*_*m*_. The addition of *g*_*m*_ limitation increased model complexity relative to ∞ *g*_*m*_ counterparts, pD's in Table 5, in most cases, with *cab* and *oil* showing exceptions with slightly reduced or similar pD's in ∞ *g*_*m*_ models. The failure of *g*_*m*_-limitation to improve model performance in all cases may have been expected given the lack of environmental stress and an attendant lack of strict plant regulation of *g*_*m*_. If water, heat and/or salinity stress were imposed, then the increased model complexity associated with dynamic *g*_*m*_ may in fact have been necessary to accurately represent the *A/C*_*i*_ response (Grassi and Magnani, 2005; Niinemets et al., 2009b; Tomás et al., 2014).

Third, the addition of temperature constraints had limited influence on model performance as in most cases temperature limited models and their counterparts were not discriminated by ΔDIC; four of six genotypes had both temperature constrained and the unconstrained alterative in there top-tier (Table 5). From an empirical perspective, this is promising as it indicates that the instrumentation and methodology used distinguished trait differences among the genotypes despite any temperature differences among trials or throughout the *A/C*_*i*_ measurement period. Temperature constraints have been universally advocated and biochemically justified for informing parameterization (Berry and Bjorkman, 1980; von Caemmerer, 2000; Bernacchi et al., 2001; Yamori et al., 2014). Trait evaluation over greater temperature ranges may identify where these two model classes (temperature constrained vs. unconstrained) differ in suitability.

### Genotype level parameterization

We found differing degrees of genotypic trait variation based on evaluation of posterior distributions revealing a hierarchical structure of photosynthetic trait variation (Figures 5–8 and Tables 6). Using an HDI percentile analysis *K*_*o(25)*_, ϕ_*J*_, θ_*J*_, and *E*_*i*_'s, other than *E*_*Vcmax*_ and *E*_*Jmax*_ did not show genotypic variability (Table 6). Lack of variability in *K*_*o(25)*_ reflects the limited mutational landscape for RuBisCO proteins (Studer et al., 2014) even while selection promotes diversification of other traits. The emergence of *K*_*c*(25)_ as variable in two models was surprising for this reason and points for the need to reconsider the prior distributions of this trait in future analysis Non-variable results also support trait conservation for temperature dependencies with the possible exception of *E*_*Vcmax*_ and *E*_*Jmax*_ (Sharkey et al., 2007). For these temperature dependencies, Medlyn et al. (2002a) used *A/Ci* curves at different temperatures to establish *E*_*i*_'s; such an approach could confirm results found here. The non-variable results for estimates of ϕ_*J*_ and θ_*J*_ can be explained potentially by the lack of light variation in the *A/Ci* dataset. At saturating light conditions variation in *J*_*max*_ would be expected while the light conditions would not serve as strong drivers of ETR response for low light traits ϕ_*J*_ and θ_*J*_ (Figures 6, 8). An analysis using a combined *A/Ci* and light response (LR) curve approach (Patrick et al., 2009) should inform estimates of ϕ_*J*_ and θ_*J*_ (Evans et al., 1993). Better integration of chlorophyll fluorescence data may also improve the models ability to identify genotypic variation in ETR traits. The degree of variation found in this population for *J*_*max*_ and *V*_*cmax*_ was striking, particularly as *J*_*max*_ showed variation beyond the data provided as prior (Figure 10) (Wullschleger, 1993). Variability in Γ^*^ puts in question the continued use of constants for describing Γ^*^, and supports the observation that complex diffusion pathways and potential environmental feedbacks complicate the estimation of Γ^*^ (Hanson et al., 2016).

Based on our analysis of this population, *J*_*max*_, *V*_*cmax*_, and Γ^*^ have a high probability of variation as multiple models described them as variable at high HDI percentiles (Figure 9 and Table 6). Although we sampled the range of extreme crop phenotypes found in *B*. *rapa* (including cabbages with high leaf allocation, turnips with dramatic root allocation, and brocoletto and oilseed types with predominant reproductive allocation), trait distinction was highest between the two RILs, which were full siblings (Figures 5–8). While the parents differ in key photosynthetic traits (Edwards et al., 2011), the even greater phenotypic difference expressed between these two RILs must arise from transgressive segregation in *WUE* (Edwards et al., 2012) and reflects either novel additive effects of allelic combinations or novel epistasis (Rieseberg et al., 1999, 2003). In contrast to the highly differentiated RIL traits, crop accessions may vary more in biomass partitioning than photosynthetic traits, reflecting the targets of selection during domestication and diversification (Edwards et al., 2016; Yarkhunova et al., 2016). The fact that phenotypes are more readily distinguished between RILs (full siblings) than among crops highlights the opportunity for genetic characterization of these traits in experimental genotypes.

Our results illustrate the need for a genotypic parameterization scheme (Figure 3) while offering targets (Figure 9) for further genetic dissection. We therefore propose genomic and transcriptomic analysis to further understanding of the factors controlling observed trait distinction among identified targets. The differentiation between RILs for many traits suggests that the existing RIL population derived from a cross between an oilseed (R500) and a rapid cycling genotype (IMB211) would be an effective one in which to begin the genetic dissection of traits underlying variation in *A*. RIL populations developed from crosses between R500 × turnip, R500 × cabbage, and R500 × brocoletto that are under development will provide additional segregating lines for the genetic dissection of *A* within this crop following a process similar to one in *Zea mays* (Dell'Acqua et al., 2015). Breeding efforts focused on the mechanisms underlying variations in *J*_*max*25_ and *V*_*cmax*25_ constitute the best targets for increasing *A* and thereby yield in agricultural crops (Long et al., 2006).

### Method limitations

The curve-fitting method of *g*_*m*_ estimation does not have the benefit of using alternative *g*_*m*_ measurement techniques based on other data types (Pons et al., 2009; Tazoe et al., 2011; Hanson et al., 2016). Estimation of *g*_*m*_ based on combined fluorometry/gas exchange methods should consider the consequences of alternative electron pathways for *A*_*J*_, because differences between linear electron transport and total electron transport may not be entirely accounted for through *g*_*m*_ alone (Yin et al., 2009). State of the art methods propose a dynamic *g*_*m*_ responding to variations in both CO_2_ partial pressure and light using variable ETR rates from chlorophyll fluorescence and/or online discrimination methods (Tazoe et al., 2011; Gu and Sun, 2014). Introduced here is a methodological approach that addresses uncertainty and enables rapid screening. Dynamic *g*_*m*_ models could be incorporated into the screening tool given appropriate data to address uncertainty associated with online discrimination techniques. A fully integrated photosynthesis model using linear electron flow and total electron flow from gas exchange and fluorometry observations coupled to online discrimination data may help resolve concerns related to *g*_*m*_ estimation (Pons et al., 2009; Tazoe et al., 2011; Gu and Sun, 2014).

Partitioning of energy between PSI and PSII (*f*) was assumed 0.5, an assumption that does not hold in all cases (Laisk and Loreto, 1996). This assumption complicates the understanding of variation in *A*_*J*;_ if the assumption is valid, then mechanics of PSII light harvesting appear to be different in *oil* relative to others, however if invalid, then *oil* may in fact have different photosystem partitioning relative to other genotypes. *f* could have been made a parameterized trait but lacking meaningful data we choose to set *f c*onstant. Finally, we lack independent validation of parameters. Many parameters (*R*_*d*_, Γ^*^, ϕ_*J*_*)* can be estimated independently through alternative gas-exchange methodologies (Laisk et al., 2002, 2007; Hanson et al., 2016), while others (*K*_*c*_, *K*_*o*_) can be evaluated using *in-vitro* methodologies (von Caemmerer et al., 1994). Such parameter validation would provide an alternate means of assessing model suitability and could be integrated into a Bayesian framework. The practical implications of proposed trait validation, including *g*_*m*_ mentioned above, on large populations remain problematic monetarily and logistically.

Finally, alternatives to DIC could be considered in future multimodel comparison studies, such as the widely Applicable Information Criteria (Watanabe, 2010). DIC relies heavily on the mean of the posterior distribution presenting some bias against posteriors with skewed distribution, potentially a problem for posteriors here including *g*_*m*_, but these alternate scoring metrics are not ready implemented in rjags currently (Watanabe, 2010; Gelman et al., 2014).

### Implications

Our modeling of phenotypic variation helps clarify how allelic variation impacts the expression of biophysical traits (Figure 1). Three improvements along the pipeline from large breeding populations to selection of genotypes with enhanced yield and stress response were identified. Two of these improvements support the use of multiple model approaches for discovering important information content not available in single model analysis. First comparison of multiple models was critical in determining differentiation of traits among genotypes. For example, the CiCc_Jm_Temp model, which is similar to a commonly utilized method (Sharkey et al., 2007), did not identify *R*_*d*_ as variable based HDI analysis, yet six of the remaining seven models did. Further, CiCc_Jm_Temp found *g*_*m25*_ to be variable at 80% HDI, the highest *g*_*m*_ variation found. Given finite resources for further investigation, our approach supports quantifying the genetic architecture of *R*_*d*_ within this population (Figure 9) while an approach solely relying on CiCc_Jm_Temp would support scrutiny of *g*_*m*_. The demonstrated uncertainty in trait estimates also supports focused model improvement and/or modified experimentation. Second, potential genotypic differences were revealed using complexity analysis, which would not have been observed in single-model analysis. Specifically, complexity analysis demonstrated ETR differences in this population as some genotypes wholly selected Jm based models while others *oil* selected an alternative ETR derivation (Table 5; Figures 5–8). Pitting competing models against one another allowed specific genotypic responses to emerge and identified model components in need of revision. Moreover, testing competing mechanistic models is superior to null hypothesis testing using frequentist statistical approaches (McElreath, 2016). Finally, the posterior trait distributions represent knowledge to be preserved as one expands models. The Bayesian updating procedures of the sensitivity analysis provides a way to codify this knowledge. Information preservation further informs our understanding of plant physiology and should embolden modelers attempting to link traits relevant to plant productivity to genes. *V*_*cmax*_*, J*_*max*_, and *g*_*m*_ are hypothesized to underline genetic variation in *A* for 13 lines of *Ozyzo sativa* (Gu et al., 2012), as was similarly shown here for *V*_*cmax*_ & *J*_*max*_. Indeed, both genotypic and evolutionarily conserved parameters have been advocated for crop models (Yin et al., 2004; Bertin et al., 2009; Gu et al., 2014). We can think of these as having a hierarchical organization in which genotypic parameters are clearly distinguished from conserved parameters. This hierarchy should be continually informed by both modeling output such as those provided here and through phylogenetic analysis of genomes when possible (Galmes et al., 2005).

## Conclusions

The integration of data from six genotypes into eight photosynthesis models allowed for a comprehensive exploration of trait space occupied by this population. We found considerable variability in key photosynthetic traits of a globally important agricultural crop while revealing a hierarchical structure of trait variation. Because photosynthesis represents one of the major processes governing plant growth and development, the genotype level screening described here using competing mechanistic models can inform our understanding of the links between observed variances and genetic controls. Bayesian methodology, emerging as a powerful tool in plant sciences, permits the explicit incorporation of prior information, propagation of uncertainty from measurements to models and offers a way to improve phenotyping methods while incorporating new data and theory.

## Author contributions

TA, BE, and CW: planned and designed experiments; TA, JP, and BE: conducted fieldwork; JP and DM: developed models and analyzed data; JP, DM, TA, BE, and CW: wrote the manuscript.

### Conflict of interest statement

The authors declare that the research was conducted in the absence of any commercial or financial relationships that could be construed as a potential conflict of interest.
